# Essential Amino Acids and Exercise Tolerance in Elderly Muscle-Depleted Subjects with Chronic Diseases: A Rehabilitation without Rehabilitation?

**DOI:** 10.1155/2014/341603

**Published:** 2014-06-09

**Authors:** Roberto Aquilani, Giuseppe D'Antona, Paola Baiardi, Arianna Gambino, Paolo Iadarola, Simona Viglio, Evasio Pasini, Manuela Verri, Annalisa Barbieri, Federica Boschi

**Affiliations:** ^1^Servizio di Fisiopatologia Metabolico-Nutrizionale e Nutrizione Clinica, Fondazione S. Maugeri, IRCCS, Istituto Scientifico di Montescano, Via per Montescano 31, Montescano, 27040 Pavia, Italy; ^2^Laboratorio Universitario per lo Studio delle Attività Motorie nelle Malattie Rare (LUSAMMR), Via Ugo Foscolo 13, Voghera, 27058 Pavia, Italy; ^3^Dipartimento di Medicina Molecolare, Università degli Studi di Pavia, Viale Taramelli 3/B, 27100 Pavia, Italy; ^4^Direzione Scientifica Centrale, Fondazione Salvatore Maugeri, IRCCS, Via Salvatore Maugeri 8-10, 27100 Pavia, Italy; ^5^Consorzio Valutazioni Biologiche e Farmacologiche, Via Luigi Porta 14, 27100 Pavia, Italy; ^6^Dipartimento di Biologia e Biotecnologie, Università degli Studi di Pavia, Via Ferrata 1, 27100 Pavia, Italy; ^7^Fondazione S. Maugeri, IRCCS, Istituto Scientifico di Lumezzane, Via Mazzini 9, Lumezzane, 25065 Brescia, Italy; ^8^Dipartimento di Medicina Legale, Scienze Forensi e Farmaco-Tossicologiche “A. Fornari”, Sezione di Scienze Farmacologiche e Tossicologiche, Università degli Studi di Pavia, 27100 Pavia, Italy; ^9^Dipartimento di Scienze del Farmaco, Università degli Studi di Pavia, Viale Taramelli 14, 27100 Pavia, Italy

## Abstract

Exercise intolerance remains problematic in subjects with chronic heart failure (CHF) and/or chronic obstructive pulmonary disease (COPD). Recent studies show that supplemented essential amino acids (EAAs) may exert beneficial effects on CHF/COPD physical capacity. The results from 3 investigations (2 conducted on CHF and 1 on COPD subjects) served as the basis for this paper. The 3 studies consistently showed that elderly CHF and COPD improved exercise intolerance after 1–3 months of EAA supplementation (8 g/d). In CHF exercise capacity increased 18.7% to 23% (watts; bicycle test), and 12% to 22% (meters) in 6 min walking test. Moreover, patients reduced their resting plasma lactate levels (by 25%) and improved tissue insulin sensitivity by 16% (HOMA index). COPD subjects enjoyed similar benefits as CHF ones. They increased physical autonomy by 78.6% steps/day and decreased resting plasma lactate concentrations by 23%. EAA mechanisms explaining improved exercise intolerance could be increases in muscle aerobic metabolism, mass and function, and improvement of tissue insulin sensitivity (the latter only for the CHF population). These mechanisms could be accounted for by EAA's intrinsic physiological activity which increases myofibrils and mitochondria genesis in skeletal muscle and myocardium and glucose control. Supplemented EAAs can improve the physical autonomy of subjects with CHF/COPD.

## 1. Introduction

Patients with chronic heart failure (CHF) and/or chronic obstructive pulmonary disease (COPD) often have reduced exercise tolerance, limiting participation in daily activities. Early onsets of fatigue and/or dyspnea are the symptoms responsible for this exercise intolerance. These symptoms are caused by peripheral mechanisms including abnormalities in skeletal muscle histology, metabolism, and function [1]. They are not caused by altered central cardiac function such as left ventricular ejection (CHF) [2] or the rate of airway obstruction (COPD) [3].

Exercise intolerance, accentuated in the elderly because of aging body changes, negatively impacts both functional and life prognosis. Indeed, it increases the risk of physical dependence, poor quality of life [4], and increased mortality rate both in CHF [5] and COPD [3]. In CHF, it is exercise intolerance and not heart function which is the most important prognostic factor [5], which furthermore is more accurate than hemodynamic/ventilator profiles at predicting outcomes [5]. Therefore, improved exercise intolerance of CHF/COPD patients is a key for maintaining the subjects' autonomy and quality of life as well as for increasing survival.

At present, treatments to improve exercise intolerance in CHF/COPD include specific pharmacological therapy and exercise training (ET). However, despite advanced pharmacological therapy, exercise intolerance still remains problematic in CHF/COPD individuals [4]. Although ET is the corner stone of cardiac and pulmonary rehabilitation (Rehab), it has not become an integral part of clinical management of patients with CHF/COPD because only very few of these patients enter a Rehab programme [6] (in Europe less than 20%). The prevalence of COPD patients who undergo any Rehab protocol is not known. Moreover, not all subjects, including less elderly patients, can sustain ET because of compromised CHF/COPD and/or of more frequent comorbidities.

Recently, a nutritional approach has emerged as a potentially useful tool to improve exercise intolerance in CHF/COPD. Some studies [7–10] have found that chronic oral supplementation of essential amino acids (EAAs) (8 g/d over 1–3 months) significantly improves exercise intolerance of elderly patients with severe CHF/COPD, living at home or admitted to a Rehab setting.

In this paper we summarize the main findings of these studies and show that the EAA mechanisms leading to improved exercise intolerance rely on the intrinsic biochemical physiology of EAAs and on changes in skeletal muscle when EAAs are provided. We believe a better understanding of more comprehensive EAA activity can contribute to future clinical research. If confirmed by larger trials, EAA supplementation could allow elderly subjects with CHF/COPD, who are not on ET programmes, to achieve a good prognostic Rehab outcome.

## 2. EAA-Induced Exercise Improvements in CHF/COPD and Their Mechanisms

There is a huge literature on the effects of acute amino acid administration on exercise performance in healthy subjects and in athletes. However, only a few studies have been carried out on the chronic use of EAAs to improve exercise intolerance in elderly subjects suffering from severe CHF/COPD and altered body composition. Preliminary results show positive effects of EAAs on exercise intolerance. In Tables 1 and 2, the amino acid mixture used in these studies and their respectively changes in certain exercise variables after chronic amino acid supplementation are reported, after evaluation from the original investigations [8–10].

### 2.1. EAAs and Exercise in CHF Subjects (Table 2)

Three investigations reported that it is possible to increase exercise capacity of patients with CHF on maximal standard therapy. In one study [8] conducted on muscle-depleted patients (arm muscle area < 5° percentile) (*n* = 44), two-month EAA treatment (8 g/d) was associated with significant improvements in work performance. Six-minute walk distance (meters) increased by 22%, being significantly more than their placebo counterparts (+4%). The improvement in physical capacity was confirmed with the bicycle exercise test (+18.7% watts versus +3.5% watts in placebo). In this test, muscle aerobic metabolism, indicated by peak oxygen consumption (mL/min/kg; peak VO_2_), increased by 10.4% in EAA subjects and 0.08% in the placebo group. At rest, lactate concentration (micromole/L) decreased by 25% in EAAs whereas it worsened in placebo CHF (+15%) indicating a reduction for the EAA group but an increase in the placebo group of muscle anaerobic processes. Insulin resistance (HOMA index) diminished by 16% in treated CHF, while it increased by 6.5% in the placebo group.

EAAs also improved nutritional status. Body weight increased in 80% of EAA subjects (+3 Kg; +4.1% baseline) and in 30% of placebo subjects (+0.4 Kg; +0.6%). This difference in distribution between EAA and control groups was significant (*P* < 0.05). Skeletal muscle mass, indicated by arm muscle area, increased by 11.8% in EAA subjects and 8.4% in control (n.s.). Given that, before the protocol started, the criterium chosen for considering EAA efficacy was the combination of an increase in body weight > 1 Kg and an increase of arm muscle area; the EAA associated changes in nutritional status were significantly higher in the treated compared to the placebo group.

The benefit of EAAs for exercise tolerance was confirmed by two investigations performed in ambulatory CHF patients. In the first study [9], elderly CHF (*n* = 15) on 3-month EAA treatment (8 g/d) improved their physical capacity at 6 min walk distance by 12% (the study had no placebo controls). In the second study [7], elderly CHF (*n* = 95) increased their exercise tolerance (bicycle exercise test) by 23% watts following 1-month EAA supplementation. In placebo subjects exercise capacity improved by 4%. The postpeak VO_2_ recovery time, the length of which is an index of anaerobic metabolism, calculated at 30% postpeak VO_2_ decline decreased by 58% in EAA treated CHF and by 14% in placebo controls. At 50% postpeak VO_2_ decline, the VO_2_ recovery time decreased by 49% in EAA subjects and by only 1% in the placebo group.

To sum up, the available studies conducted on elderly subjects with CHF consistently reported significant improvements of exercise intolerance following chronic EAA supplementation. The plausible mechanisms included improved muscle aerobic metabolism energy-producing and nutritional status and reduced insulin resistance.

### 2.2. EAAs and Physical Capacity in COPD Subjects (Table 2)

Similar results were also observed in ambulatory COPD population (*n* = 60) [10]. These patients were sarcopenic (bioimpedance analysis measure) and were on long-term oxygen therapy. Three-month EAA supplementation (8 g/d) improved their physical capacity in terms of number of steps/day by 78.6% whereas subjects on placebo tended to have step diminution (−7.8%). EAA treatment improved muscle efficiency in aerobic energy production as suggested by the 23% reduction in resting plasma lactate levels compared with the pre-EAA treatment period. In contrast, plasma lactate increased by 13% in the placebo group. It is interesting to note that both the time courses and the changes of plasma lactate concentrations were similar for muscle-depleted CHF [8] and sarcopenic COPD. This indirectly confirms the fact that the myopathy of CHF and COPD patients shares several metabolic alterations [1].

Again, similar to the CHF study, EAAs also improved patient nutritional status. Body weight increased by an average of 5.5 kg (+10.3% baseline) of which 3.66 kg was fat free mass (FFM). In controls, body weight decreased by 3.5% but FFM increased by 0.6% (+3.8 kg). Probably in this latter group, the overtime energy intake was inadequate and/or there was an increase of body water. Indeed, in treated subjects but not controls, physical capacity and FFM improvements were associated with increases in muscle strength (handgrip) (+1.6 kg; +7.4%), serum albumin concentrations (+4.28 g/L), cognitive function (+1.62 scores at Minimental Test Examination), and quality of life perception (St. George's Respiratory Questionnaire, −2.7 scores). Like the CHF, improved muscle aerobic metabolism and nutritional status accounted for the improved exercise capacity of patients.

In addition, EAA treatment induced pluridistrict extramuscular anabolic activity in COPD subjects including that in the cerebral region, which is very sensitive to amino acid activity [27].

### 2.3. Other Interventional Studies Indirectly Suggest Improvement in Exercise Intolerance after EAA Supplementation

Given that muscle mass and, above all, muscle strength are good predictors of physical capacity, their increase after EAA supplementation (8 g/d) indirectly suggests improved physical performance.

Nutritional status significantly improved following EAA treatment (3 months, 8 g/d) in cachectic COPD (dual X-ray absorption measure) [11]. Patients' weight increased by 3.8 ± 2.6 kg in the EAA group and −0.1 ± 1.1 kg in the placebo one. FFM increased in 69% and 15% of EAA and control patients, respectively. FFM average increase was 1.5 ± 2.6 kg in EAA subjects and −0.1 ± 2.3 kg in controls.

In another study, EAA supplementation positively affected muscle strength in healthy elderly people [12] and in institutionalized old individuals [13]. In this study, EAAs subjects also significantly improved daily activity, mininutritional assessment score, depressive symptoms, and quality of life.

The effect of EAAs on insulin resistance [8] was confirmed in a cohort of elderly type 2 diabetics who showed long-term (60 weeks) better glucose control [14]. Interestingly, in another population of elderly diabetics, EAA supplementation improved cardiac performance as indicated by increased left ventricular ejection fraction [9]. This was also reported in healthy elderly individuals after 3 months of EAA treatment [12].

## 3. EAA Mechanisms Leading to Improved Exercise Intolerance Rely on the Intrinsic Biochemical EAA Activities and Skeletal (Cardiac) Muscle Changes following Chronic EAA Treatment (Table 3)

Improved muscle aerobic metabolism, prevalence of muscle anabolic processes, and reduction of insulin resistance (the latter in CHF) are the main EAA mechanisms explaining increased exercise tolerance in both CHF and COPD subjects on maximal standard therapy [7–10]. These mechanisms mutually influence each other and, in turn, rely on the intrinsic biochemical EAA properties and profound skeletal (and cardiac) muscle changes following chronic EAA supplementation documented by a number of experimental studies. Here the mechanisms will be discussed separately for more clarity.

### 3.1. Improvements in Muscle Aerobic Energy Production (Table 3)

Both CHF and CODP patients at rest are biochemically characterized by impaired formation of muscle energy availability (adenosine three phosphate-ATP and creatine phosphate-CP compounds) [1]. Adequate cell energy availability is essential to develop muscle strength and protein turnover (protein synthesis and proteolysis).

EAAs are the substrates which can enhance muscle aerobic metabolism because they act as fuel for Krebs cycle (tricarboxylic cycle acid: TCA), promoting mitochondria biogenesis and tackling the negative effects of insulin resistance on the TCA cycle. Indeed, EAAs can enter the TCA cycle at various levels and can be used as an alternative fuel for producing energy [28], as their activities are independent of insulin [21]. EAA supplements normalized the ATP content and production rate in aged rat* gastrocnemius* muscle similar to those of adult rats [15].

Furthermore, EAAs upregulate mitochondria synthesis and increase their volume and cellular density [18, 20]. Interestingly, mitochondria biogenesis by EAAs is also upregulated in cardiac muscle [18]. In middle-aged mice, the essential branched-chain amino acid (BCAA) supplement increased mitochondrial biogenesis and sirtuin-1 expression (a member of sirtuin family linked to the life span extension), in cardiac and skeletal muscle, accompanied by enhanced physical performance [19].

All of these experimental studies suggest that chronic EAA supplementation both directly and indirectly increases muscle cell availability in high energy compounds indispensable for improving work performance and muscle strength in elderly individuals. This is independent of whether they are healthy [12] or affected by COPD [10] or CHF [9].

### 3.2. Improvements in Muscle Insulin Resistance (Table 3)

EAA supplementation can make muscle aerobic metabolism more efficient by reducing insulin resistance [14]. EAAs directly upregulate insulin-receptor synthesis and its autophosphorylation [14]. Less insulin resistance diminishes the block of the cell pyruvate dehydrogenase complex caused by insulin resistance [8] and by circulating inflammatory cytokines in both CHF and COPD. Better activity of this enzymatic complex improves energy formation from glucose oxidation. Consequently, the lactate ⇄ pyruvate reaction shifts towards the right [8].

In induced diabetic rat hearts, long-term oral EAA supplementation increased mitochondrial cytochrome c oxidase and NADH-H activities and significantly shifted the ventricular myosin heavy chain pattern towards a faster phenotype [16]. Both in diabetic and in healthy mice, EAA supplements modulate the skeletal muscle redox state by improving the antioxidant defense system as shown by increased superoxide dismutase (SOD) expression and simultaneous decrease in heat shock proteins [17].

EAAs may reduce insulin resistance by lowering the circulating cytokine tumor necrosis factor alpha. This has been demonstrated in sarcopenic elderly subjects [14].

### 3.3. Improvements in Skeletal (and Cardiac) Muscle Anabolism

Improved nutritional status observed in muscle-depleted patients [8, 10, 11, 13] clearly indicates that even subjects with severely reduced FFM and on maximal standard therapy can improve their nutritional status when supplemented with physiological amounts of EAAs. This is not surprising given that the physiological role of EAAs is both to increase protein synthesis and to decrease protein breakdown.

Chronic supplementation of essential BCAA leucine upregulates protein synthesis in skeletal muscle, adipose tissue, and liver [21] by enhancing activity synthesis of proteins involved in mRNA translation. Particularly relevant to elderly CHF/COPD patients, leucine (and other EAAs) acts as a nutritional signaling molecule, quite independent of insulin [21]. However, amino acids regulate insulin signaling via mTOR nutrient signaling [29] and their adequate availability is indispensable for insulin to exert its anabolic activity [29]. On the other hand, reduced amino acid availability lowers mTOR activation even with improved insulin signaling. This was documented in healthy and in insulin resistant human skeletal muscle [29]. Even small doses of exogenous EAAs can stimulate muscle protein metabolism [22].

Another pathway by which EAAs induce protein synthesis is by upregulating the hepatic production of the anabolic hormone insulin-like growth factor 1 (IGF-1). This hormone is dependent on EAA availability in the blood and acts only when there is adequate EAA availability. A study reported that 7.5 g EAAs (an amount very close to that used in the studies on EAAs and exercise intolerance) increased IGF-1 levels [25].

The fact that EAAs stimulate muscle protein metabolism in healthy elderly subjects [23] is of practical importance for elderly CHF/COPD individuals needing nutritional supplementation. This is because pharmaceutical formula reflecting the composition of a standard meal or high protein diet fails to increase muscle mass and strength as well as protein synthesis in the elderly [22]. EAA can induce net protein synthesis by reducing muscle protein breakdown. In human studies, the efficiency of protein use is due to the reduced sensitivity of proteolysis rather than any changes in protein synthesis [24].

Particularly interesting is the effect of leucine on protein metabolism in the myocardium. Leucine is the only amino acid that can inhibit protein degradation in the myocardium [30] and its inhibitory effect is mediated by extracellular leucine [30]. On the other hand, BCAA transamination in the heart is 3 times higher than in peripheral skeletal muscles [30]. The possible effect of EAAs on myocardium could explain the improved left ventricular dysfunction in healthy elderly individuals [9] and the faster exercise VO_2_ recovery time in elderly subjects with CHF [7, 26].

In synthesis, experimental and human investigations have shown that chronic EAAs supplementation may increase and make the muscle aerobic energy production more efficient and increase skeletal muscle mass/strength and mitochondria number and function. It has been reported [31] that 75% of the body's nitrogen requirement is supplied by leucine, isoleucine, valine, threonine, and lysine, all amino acids contained in the nutritional mixture used here. It is interesting to note that the mixture's amino acids were formulated in reciprocal stoichiometric ratios, specifically both to match metabolism energy needs and to maintain protein synthesis [31].

The histological and biochemical changes associated with EAA supplementation overlap those induced by ET Rehab (Table 4). It is also interesting to note that the EAA dose (8 g/d) is the amount contained in higher protein quality foods usually consumed by both healthy and ill subjects (Table 5) [32].

To understand the importance of the reciprocal stoichiometric ratios of the amino acids in the mixture [31], very recently an experimental study [33] reported that excessive neurotransmitter serotonin derived from tryptophan and noradrenergic activity from tyrosine may negatively impact the physical activity status particularly in sedentary animals. Moreover excessive tryptophan consumption can reduce physical activity by inducing central fatigue [34]. Interestingly, excessive tryptophan may also limit the blood-brain-barrier passage of leucine, isoleucine, valine, and tyrosine, therefore reducing their availability within the brain structure [35] and contributing to further limiting physical capacity.

## 4. Conclusions

Elderly patients with CHF/COPD when supplemented with EAAs not only may improve exercise intolerance but also can achieve some prognostic outcomes typical of ET Rehab. Obviously, EAAs does not substitute comprehensive Rehab but may be very useful for elderly subjects with CHF/COPD who, for various reasons, cannot undergo ET Rehab.

## Figures and Tables

**Table 1:** Nutritional composition of an individual packet of supplementation, containing 4 g of an amino acid mixture, used in the clinical studies* reported in the current investigation [7–14].

Kcal	35.3
KJ	149.9
Total amino acids, of which	4 g
L-Leucine	1250 mg
L-Lysine	650 mg
L-Isoleucine	625 mg
L-Valine	625 mg
L-Threonine	350 mg
L-Cysteine	150 mg
L-Histidine	150 mg
L-Phenylalanine	100 mg
L-Methionine	50 mg
L-Tyrosine	30 mg
L-Tryptophan	20 mg

*Treated patients were given 2 packets daily (8 g essential amino acids).

**Table 2:** Changes in exercise variables observed after EAA supplementation in subjects with chronic heart failure or chronic obstructive pulmonary disease.

Exercise variables	Disease	Treatment duration	Changes (% pretreatment)	
			Placebo	EAAs
Mechanical work				
6 min WT (meters)	CHF	12 weeks	n.d.	+12 [9]
	CHF	8 weeks	+4	+22 [8]
Cycle ergometer (watts)	CHF	8 weeks	+3.5	+18.7 [8]
	CHF	4 weeks	+4	+23 [7]
Steps (number/day)	COPD	12 weeks	−7.8	+78.6 [10]
Metabolic variables during cycling				
(a) Aerobic metabolism:	CHF	8 weeks		
VO_2_ peak (mL/Kg/min)			+0.08	+10.4 [7, 8]
(b) Anaerobic metabolism:	CHF	4 weeks		
VO_2_ recovery time (mL/Kg/min)				
(i) At 30% postpeak decline			−14	−58 [7]
(ii) At 50% postpeak decline			−1	−49 [7]
Resting plasma lactate (*μ*mol/L)	CHF	8 weeks	+15	−25 [8]
	COPD	12 weeks	+13	−23 [10]
Resting insulin resistance (HOMA index)	CHF	8 weeks	+6.5	−16 [8]

WT: walking test; VO_2_: oxygen uptake; CHF: chronic heart failure; COPD: chronic obstructive pulmonary disease; n.d.: not determined.

**Table 3:** EAA physiological activities and histological-biochemical findings from *in vivo* and human studies following chronic EAA supplementation, explaining the EAA mechanisms in improving exercise intolerance in CHF/COPD.

Mechanisms and measures in CHF/COPD	Physiological activities	Findings from experimental and human studies	
		Biochemistry	Histology
Increased aerobic metabolism WT Steps/day VO_2_ peak Time VO_2_ peak to baseline Resting plasma lactate levels	EAAs used as fuel for TCA cycle	↑ ATP production and ↑ cell ATP availability [15] Shift of ventricular MHC from *β* to *α* type [16] ↑ COX and NADH^+^ activities [16] ↑ SOD [17]	↑ Mitochondria number: +310% skeletal muscle +40% myocardium [18] ↑ 28% increased mitochondria volume [18] ↑ Mitochondrial biogenesis and sirtuin 1 expression in cardiac and skeletal muscle [19] ↑ Vsar/Vtot fiber ratio [20]

Improved nutritional status FFM Body weight Muscle strength Serum albumin levels	↑ Protein synthesis [21–23] ↓ Proteolysis [24] ↑ IGF-1 expression [25]	↓ TNF alpha/IGF 1 ratio [14]	↑ 40% myofibrils of quadriceps muscle [18] ↓ Muscle fibrosis [18] ↑ Type II A fibers [20] ↑ Cross-sectional area of skeletal muscle fibers [20]

Reduced insulin resistance	Upregulated insulin-receptor synthesis and its autophosphorylation [14]	↓ HOMA index [14] ↓ Fasting insulin levels [14] ↓ Fasting blood glucose [14] ↓ TNF alpha/IGF 1 ratio [14]	

WT: walking test; VO_2: _oxygen consumption; FFM: fat free mass; TCA: tricarboxylic cycle acid; ATP: adenosine triphosphate; COX: cytochrome oxidase; NADH: nicotinamide adenine dehydrogenase; SOD: superoxide dismutase; IGF-1: insulin-like growth factor-1; TNF: tumor necrosis factor; Vsar/Vtot: volume of sarcomeres/total volume ratio.

**Table 4:** Some similarities between the effects of exercise therapy and those following EAA supplementation.

Measures	Exercise therapy [1, 5]	EAA supplementation
Exercise capacity		
Maximal oxygen uptake	Increased	Increased
Six-minute walk distance	Increased	Increased
Anaerobic threshold	Increased	Increased*
Maximal incremental exercise duration	Increased	Increased
Resting ejection fraction	Unchanged or slight increase	Slight, significant increase
Resting lactate production	n.d.	Reduced
Muscle structure and function		
Muscle cross-sectional area	Increased	Increased^∧^
Muscle fiber size	Increased	Increased^∧^
Number of type I muscle fibers	Increased	Increased^∧^
Mitochondrial numbers	Increased	Increased^∧^
Mitochondrial *cristae* density	Increased	Increased^∧^
Muscle dynamic strength	Increased	Increased^∧^
Muscle fatigability	Reduced	Reduced^∧^

*Inferred from improved recovery time of maximal oxygen uptake to baseline value [26]; ^∧^in animals [15–20].

n.d.: not determined.

**Table 5:** Amount of some types of high quality protein foods containing the same amount of essential amino acids (8 g) as the pharmaceutical formula used in the studies on exercise intolerance.

Food	g
Lean beef meat	97
Chicken (breast)	74
Mortadella	131
Ham	79
Cheese (average of 6 types)	105
Canned tuna fish	74
Trout	153
Codfish	97
Eggs	138
Whole milk	480
